# Relationships Between Breast Edema and Axillary Lymph Node Metastasis in Breast Cancer

**DOI:** 10.3390/diagnostics15111300

**Published:** 2025-05-22

**Authors:** Derya Deniz Altıntaş, Gul Esen Icten, Füsun Taşkın, Cihan Uras

**Affiliations:** 1 Department of Radiology, Health Science University Diyarbakır Gazi Yaşargil Tranining and Research Hospital, 21070 Kayapınar, Diyarbakır, Türkiye; deryadenizaltintas@gmail.com; 2 Senology Research Institute, Acıbadem Mehmet Ali Aydınlar University, 34638 Maslak, İstanbul, Türkiye; fusun.taskin@acibadem.edu.tr (F.T.); cihan.uras@acibadem.edu.tr (C.U.)

**Keywords:** axillary metastasis, breast cancer, breast MRI, edema, prognostic biomarker

## Abstract

**Background/Objectives:** To investigate the association between MRI features of primary breast cancers with axillary status, aiming to identify possible imaging biomarkers. **Methods:** Patients diagnosed with breast cancer between 2021 and 2023 in our clinic were retrospectively evaluated, and those that presented as mass lesions on preoperative MRI examinations (*n*: 123) were included in the study. Patients with and without metastatic axillary lymph nodes (mALN) were compared in terms of breast density, background parenchymal enhancement, tumor size, location in the breast, distance from the skin, patient age, presence of edema, multiple foci, histopathological type and molecular subtype of tumors. In multifocal/multicentric cases, the largest lesion was taken into consideration. Prepectoral and subcutaneous edema were considered diffuse edema, while perilesional edema was considered focal edema. MannWhitney U/Student-*t* test, Chi- square/Fischer Exact tests and logistic regression analysis were used for statistical analyses as appropriate. **Results:** Axilla was positive in 88 patients. There was a statistically significant difference in terms of edema, age, molecular subtype, Ki-67 index, number of lesions, tumor size, and laterality between the two groups (*p* < 0.05). Univariate logistic regression analysis showed that all included variables were statistically significant (*p* < 0.05). Multivariate logistic regression analysis revealed that presence of edema (OR: 2.46 CI; 1.11–5.48, *p* = 0.027) and multiple lesions (OR: 1.86 CI; 1.01–3.43, *p* = 0.046) were significantly associated with mALN. There was no significant difference between peritumoral edema and diffuse edema. **Conclusions:** Our study showed a statistically significant relationship between the axillary status and the presence of edema and multiple tumoral lesions on MRI. These findings have a potential to serve as prognostic imaging biomarkers for predicting the presence of mALN. Further studies with larger case series are needed to support our findings.

## 1. Introduction and Aim

Determining the status of the axillary lymph nodes [ALN] plays a crucial role in developing appropriate and personalized treatment programs in patients with breast cancer [1]. Invasive techniques, such as sentinel lymph node biopsy and axillary lymph node dissection [ALND], are commonly employed to assess ALN status. However, in approximately 70% of cases, no metastatic lymph nodes are detected, thus leading to unnecessary complications in some patients [2]. Moreover, determination of axillary status before neoadjuvant chemotherapy [NAC], and need for ALND after therapy depends solely on the initial imaging findings and guided biopsies, which may sometimes give false negative results. PET-CT is a useful diagnostic method for the evaluation of the axilla, but it can give false negative as well as false positive results. It is usually reserved for advanced cases, although we know that very small tumors may sometimes metastasize to the axilla.

MRI provides valuable insights into both morphological and functional characteristics of breast tumors [3,4]. Although its role in characterization of breast tumors is well known, it is relatively limited in the evaluation of the axilla. Recent studies have investigated the relationship between axillary status and MRI features and clinicopathological characteristics of the primary tumor. Some independent risk factors potentially associated with metastatic axillary lymph nodes have been identified in these studies, such as tumor size, localization, multifocality, presence of prepectoral edema, ADC values, molecular subtypes, and lymphovascular invasion [5,6,7]. Peritumoral edema is thought to result from the release of inflammatory cytokines, which increase vascular permeability and lead to transudation of fluid into the extracellular space surrounding the tumor tissue. In contrast, prepectoral and subcutaneous edema are believed to occur due to obstruction of lymphatic drainage in the axillary and subcutaneous tissues by tumor cells [8]. A recently published meta-analysis demonstrated a significant association between peritumoral edema and lymph node metastasis. However, to date, no study has specifically investigated the relationship between different types of edema and lymph node metastasis [9]. Utilizing imaging to accurately predict the presence of mALN could enable patients to receive the most appropriate non-invasive treatment. However, studies have focused on different criteria; data are limited and do not yet provide clear information or consensus on this matter.

The purpose of this study was to further investigate the relationship between preoperative breast MRI findings, particularly presence and types of edema, and metastatic axillary lymph nodes [mALN] in patients diagnosed with breast cancer, in order to identify imaging findings that could alert us to the presence of axillary involvement, allowing us to make better treatment decisions.

## 2. Materials and Methods

Our study was conducted retrospectively after obtaining approval from the hospital’s Ethics Committee (217/25 November 2022). Hospital records revealed that 255 patients diagnosed with breast cancer at our hospital between January 2021 and December 2023 had preoperative MRI examinations. The patients whose MRI images were available in the PACS system were included in the study. All MRI examinations were performed before needle biopsy. Male patients, patients whose breast MRI technique were suboptimal, who were previously treated for breast cancer, and who received chemotherapy before the MRI examination were excluded. For this study, only patients with tumors presenting as mass lesions were investigated, and therefore patients with non-mass lesions were also excluded.

The clinical features, immunohistochemical results, and MRI findings of the remaining 123 patients were evaluated retrospectively. ALN status was determined by sentinel lymph node and/or axillary dissection results in patients who underwent surgery, and by core-needle biopsy results in patients who received neoadjuvant chemotherapy. Informed consent was obtained from all patients in accordance with the Helsinki Declaration [10].

### 2.1. MRI Evaluation Protocol

MRI examinations were performed using a 1.5T GE Optima 360 [General Electric Medical System, Milwaukee, WI, USA] device, with patients in the prone position, in axial and sagittal planes, using an IV contrast agent [0.1 mL/kg Gadovist^®^, Bayer Schering Pharma AG, Berlin, Germany]. The standard MRI protocol included axial 2-D T2W short tau inversion recovery [STIR], turbo spin echo [TSE], axial T2W TSE, sagittal FSE T2 FatSat-R/L, axial diffusion-weighted imaging [DWI], axial pre-contrast T1W, post-contrast dynamic non-fat-suppressed axial T1W, post-processing dynamic axial T1W subtraction and contrast-enhanced sagittal spectral attenuated inversion recovery [SPAIR] sequences. MRI images were evaluated by a single radiologist with 5 years of experience in breast imaging using the BIRADS lexicon [5th edition] [4]. The images were reviewed without knowledge of the clinical findings, other radiological tests, or pathology results. Recorded features included parenchymal density [A–D], background enhancement [minimal, mild, moderate, marked], lesion size [largest diameter], lesion laterality [right, left], localization within the breast [quadrant: inner, outer, central; upper, lower, central], lesion depth [anterior, central, posterior, extensive], number of lesions [unifocal, multifocal, multicentric, diffuse], distance from the skin, presence and type of edema [perilesional, diffuse], diffusion restriction, and necrosis. Both prepectoral and subcutaneous edema were included in the diffuse edema category. The distance from the skin was measured as the closest distance on axial images. Diffusion restriction was assessed visually due to the lack of quantitative ADC values. In cases with multiple lesions, the characteristics of the largest tumor were evaluated.

### 2.2. Histopathological Evaluation of Tissue Samples

Immunohistochemical analyses were performed for ER, PR, HER2 expression, and Ki-67 index. HER2 expression was scored as 0, 1+, 2+, or 3+ through immunohistochemical analysis. Scores of 0 and 1+ were considered HER2-negative, while a score of 3+ was considered HER2-positive. For cases with HER2 2+ expression, HER2 status was determined by fluorescence in situ hybridization [FISH] to assess gene amplification. Ki-67 index cut-off value was taken as 14%.

Based on the immunohistochemical staining characteristics and Ki-67 proliferation index, lesions were classified into molecular subtypes. Patients with positive ER and/or PR receptors were considered hormone receptor [HR] positive and classified into the Luminal group. HR [+] and HER2 [−] lesions with a Ki-67 proliferation index below 14% were classified as Luminal A, while HR [+] lesions with a Ki-67 proliferation index above 14% or with HER2 positivity were classified as Luminal B. HR [−] and HER2 [+] lesions were classified as “HER2-enriched”, and HR [−] and HER2 [−] lesions were classified as “Triple negative” [11].

### 2.3. Statistical Analysis

All statistical analyses were performed using IBM SPSS 24.0. Continuous variables were presented as mean and standard deviation if they followed a normal distribution, and as median and interquartile range if they did not. Categorical variables were presented as numbers and percentages. Patients were divided into two groups based on the presence or absence of metastatic ALN. Depending on the distribution of numerical variables, the Mann–Whitney U test or Student’s *T*-test was used to compare groups, while the Chi-square or Fisher’s Exact test was used for nominal and categorical variables. Variables with a *p*-value of <0.05 after the analysis were included in a univariate logistic regression analysis. Variables with a *p*-value of <0.05 in the univariate logistic regression analysis were then used to create a multivariate logistic regression model. In this model, adjusted odds ratios [OR] and 95% confidence intervals [CI] were reported, and a *p*-value < 0.05 was considered statistically significant.

## 3. Results

The median age of the 123 patients included in the study was 46 (40–56). Histopathologic evaluation revealed that 108 patients had invasive ductal carcinoma, 5 had invasive lobular carcinoma, and 10 had other cancer types. mALN were detected in 88 patients (71.5%). Mean lesion size was 28 (17–43) mm.

**Figure 1:**
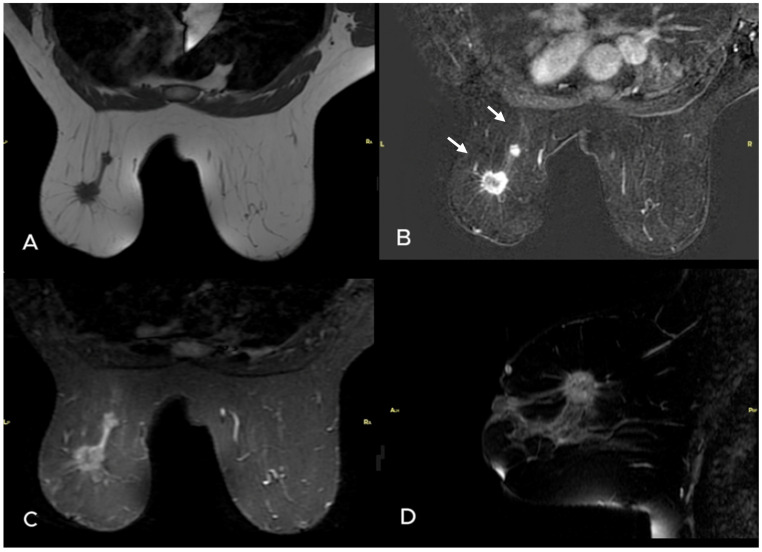
A 38-year-old woman presented with nipple retraction in the left breast. Unenhanced (**A**) and subtracted (**B**) MR images revealed 2 masses. The largest had spiculated margins and demonstrated peripheral enhancement, while the second one was well circumscribed and lobulated and demonstrated homogeneous enhancement (white arrows). Axial and sagittal TSE STIR (**C**,**D**) images showed no significant edema. Core needle biopsy results of both masses were invasive ductal arcinoma, and ER was 70%, PR was 95%, HER2 was 2 (+) (FISH (−)), and Ki-67 index was 20% for the larger mass. Postoperative pathology revealed 2 metastatic lymph nodes in the axilla.

**Figure 2:**
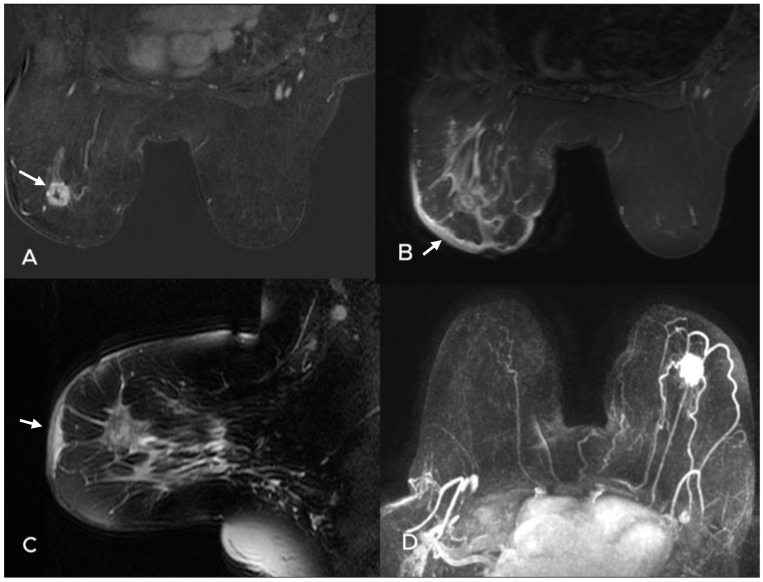
A 51-year-old woman with redness and thickening of the skin in the left breast. Preoperative MRI examination revealed a 2.5 cm round mass with irregular margins in the upper outer quadrant of the left breast. It demonstrates heterogeneous enhancement on axial substracted image (**A**), and subcutaneous edema on axial and sagittal STIR TSE images (**B**,**C**) (white arrows). A round-shaped lymph node is also seen in the axilla on the sagittal T2W (**C**) and axial maximum intensity projection images (**D**). Pathology: IDC, ER: Low positive 1–10%, PR: Negative, HER2: Score 3/++++, Ki67: 55% Core needle biopsy of the lymph node: metastasis.

**Figure 3:**
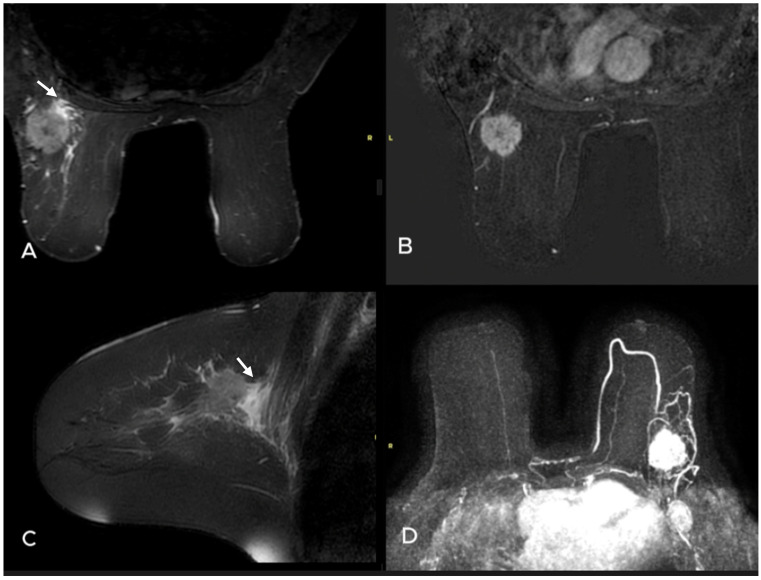
A 49-year-old woman presented with a palpable mass in the left breast. T2W (**A**) and subtracted dynamic (**B**) MR images revealed a round mass with irregular margins that showed heterogeneous enhancement. There was prepectoral and subtle perilesional edema on the axial (**A**) and sagittal (**C**) STIR TSE images (white arrows). Maximum intensity projection image also demonstrates a pathologic lymph node in the axilla (**D**). Pathology: Invasive ductal carrcinoma ER: 90%, PR: 50% C-erb B2 score: 0, Ki-67 proliferation index 40%, core-needle biopsy of the axilla: metastasis.

Patients were divided into two groups based on the presence or absence of mALN. The groups were compared according to clinical, pathological, and MRI findings [Table 1]. The median age of the group with mALN (45 (39–54)) was significantly lower than that of the non-metastatic group (48 (41–63)) (*p* = 0.042). mALN was more frequently observed in mass lesions located in the right breast (51% vs. 37.1%; *p* = 0.037). The Ki-67 levels (30 (15–43)) were significantly higher in the mALN group compared to the non-metastatic group (15 (10–25)) (*p* = 0.002). The distribution of molecular subtypes in the mALN group was as follows: Luminal A 19.3%, Luminal B 52.3%, HER-2 enriched 14.8%, and Triple-negative 13.6%. In the non-metastatic group, the distribution was 40.0%, 48.6%, 8.6%, and 2.9%, respectively, [*p* = 0.016]. Axillary metastases were more frequently detected in patients with multifocal/multicentric disease [Figure 1]. In the metastatic group, 25.0% had multifocal and 23.9% had multicentric disease, while 9.1% had diffuse involvement. On the other hand, in the non-metastatic group, percentages of patients with multifocal, multicentric and diffuse disease were 17.1%, 5.7%, and 2.9%, respectively [*p* = 0.001]. The median tumor size in the mALN group (32 mm (21–43)) was significantly larger than those in the non-metastatic group (20 mm (17–30)) (*p* < 0.001). Peritumoral and diffuse edema were more frequently observed in the mALN group compared to the non-metastatic group (peritumoral edema 48.9% vs. 40.0%, diffuse edema 35.2% vs. 11.4%; *p* < 0.001) [Figure 2 and Figure 3]. There was no statistically significant difference between the two groups in terms of the median distance of the lesion from the skin, breast density, severity of background parenchymal enhancement, presence of necrosis or diffusion restriction [Table 1].

The distribution of anatomical locations of the largest tumor is summarized in Table 2. No significant differences were found between the groups regarding Quadrant 1 [inner, outer, central], Quadrant 2 [upper, lower, central], or based on lesion depth [anterior, posterior, central, diffuse] [*p* = 0.58, *p* = 0.44; *p* = 0.35].

Variables that showed significant differences in the univariate analysis [*p* < 0.05], including age, right breast localization, Ki-67 level, molecular subtype, presence of edema, number of lesions, and tumor size, were included in a multivariate logistic regression analysis. The effect sizes were expressed as unadjusted odds ratios [OR] with 95% confidence intervals [CI], and a *p*-value < 0.05 was considered statistically significant. Significant variables included age [OR 0.96, 95% CI: 0.93–0.99, *p* = 0.023], laterality [right vs. left] [OR 0.42, 95% CI: 0.19–0.96, *p* = 0.039], edema [OR 3.19, 95% CI: 1.71–5.95, *p* < 0.001], multiple foci [OR 2.31, 95% CI: 1.34–3.98, *p* = 0.003], size [OR 1.05, 95% CI: 1.01–1.09, *p* = 0.004], molecular subtype [OR 2.05, 95% CI: 1.19–3.51, *p* = 0.009], and Ki-67 value [OR 1.02, 95% CI: 1.01–1.04, *p* = 0.022]. In the multivariate logistic regression analysis, the presence of peritumoral and diffuse edema [adjusted OR, 95% CI: 2.46, 1.11–5.48, *p* = 0.027] and multiple foci [adjusted OR, 95% CI: 1.86, 1.01–3.43, *p* = 0.046] were found to be statistically significant factors associated with the presence of mALN [Table 3]. No significant difference was found between localized peritumoral edema and diffuse edema in the multivariate logistic regression model [OR 0.66, 95% CI: 0.16–2.62, *p* = 0.55].

## 4. Discussion

Our study found a statistically significant association between the presence of edema and mALN in breast cancer, irrespective of the type of edema. This study is one of the few that have investigated the relationship between edema and mALN. Additionally, consistent with the literature, we observed a statistically significant relationship between mALN and the presence of multiple tumor foci [multifocal or multicentric].

Imaging findings are crucial for determining treatment approaches and creating personalized treatment plans for breast cancer [12]. Recent studies focus on identifying imaging biomarkers that could determine risk and impact prognosis during treatment [13]. Various studies have explored MRI’s ability to predict mALN, using differing parameters and reaching differing results. For example, a study aimed at creating a nomogram to predict mALN based on MRI features and clinicopathological characteristics reported that tumor size, location, multifocality, ADC value, and lymphovascular invasion could be independent risk factors for mALN [5]. However, we did not find a significant relationship between tumor size and mALN in the multivariate analysis. It is important to consider that a large tumor might develop from a slowly progressing disease, while a small tumor might be aggressive, suggesting tumor size should be assessed in conjunction with other factors. Additionally, our results did not show a significant relationship between diffusion restriction and mALN. However, we did not look at ADC values. They have been reported to correlate with mALN in some studies, but results are inconsistent [14,15]. Recently, Surov et al. showed that ADC values could not predict molecular subtypes or mALN in invasive breast cancers [14].

Dietzel et al. reported that early contrast washout, heterogeneous enhancement, irregular margins, skin thickening, necrosis, and edema were closely associated with mALN [16]. Our study found a significant association between edema and mALN but no significant relationship with necrosis, possibly due to the limited number of cases with necrosis. Currently, edema is not considered a significant prognostic factor in national and international guidelines [ACR and AIOM] [17]. However, peritumoral edema has been identified as a high-risk marker in the Kaiser scoring system [18]. Some studies have shown a correlation between breast edema and lymphovascular invasion and tumor aggressiveness [8,19]. Therefore, it is suggested as a potential prognostic factor. Cheon et al. reported that peritumoral edema in preoperative breast MRI was associated with disease recurrence and could aid in prognostic evaluation in invasive breast cancer [20]. Another study proposed a negative correlation between peritumoral edema and disease-free survival after neoadjuvant chemotherapy in triple-negative breast cancer [21]. Uematsu et al. found an association between edema and lymphovascular invasion, with high-grade lymphovascular invasion significantly related to prepectoral edema in breast cancer [7]. Lymphovascular invasion is a prognostic factor responsible for lymph node metastasis [22]. A recent study suggested that peritumoral edema, characterized by large size, high tumor grade, and high Ki-67 values, is associated with biologically aggressive non-luminal breast cancers and should be considered a valid additional prognostic tool [23].

Uematsu et al. classified malignancy-associated edema into peritumoral, prepectoral, and subcutaneous edema based on etiology and localization [21]. The mechanisms underlying peritumoral edema remain unclear, but it is believed to result from proteolysis and neoangiogenesis, with inflammatory cytokines increasing vascular permeability and fluid transudation in the extracellular space surrounding the tumor [24]. Prepectoral edema is associated with tumor cells in retro-mammary spaces. Blockage of axillary lymphatic drainage due to carcinoma activates mammary collaterals and prepectoral lymphatic drainage [25,26]. Subcutaneous edema arises from the obstruction of lymphatic drainage in subcutaneous tissue by tumor emboli [27]. Therefore, peritumoral edema is considered the mildest form, prepectoral edema is generally preceding subcutaneous edema, and subcutaneous edema is viewed as the “final stage of malignancy-associated breast edema” [21]. In our study, although presence of edema was significantly associated with axillary metastasis, we did not find a significant difference between the types or extent of edema. A recent study has also indicated that while edema might be a predictor of tumor biological tumor aggressiveness, no significant correlation was found between edema type and lymphadenopathy [28]. Gemici et al. reported that peritumoral edema was associated with lymphovascular invasion, tumor size, and Ki-67 level, while prepectoral edema did not show a significant relationship with other parameters, and widespread edema correlated with tumor size [29].

The second statistically significant factor in the multivariate analysis in our study was presence of multiple tumor foci. With the increasing use of preoperative MRI and advanced pathology techniques, multifocal and multicentric breast cancer cases are being diagnosed more frequently [30,31]. Due to varying definitions and sensitivities of diagnostic methods, the incidence rates of multiple tumors have been reported on a wide scale, ranging from 9% to 75% [32,33]. MRI’s high sensitivity for detecting additional cancer foci is a major advantage, and it has been reported that MRI can detect additional lesions in 10–30% of patients [34]. On the other hand, Miller et al. report that additional lesions were present in 39 out of 81 patients [48.1%] in a more recent study [35]. Similarly, 48.9% of the patients had multiple lesions in our study. Tumor size is an important determinant of mALN and is believed to be directly correlated with mALN [36,37]. It is a key prognostic feature and a quantitative imaging biomarker. It was one of the significant criteria in univariate analysis in our study too, but not in the multivariate analysis. This discrepancy may be due to the increased overall mean tumor size in our cohort as well as the high number of patients with multifocal/multicentric disease. Multifocality and multicentricity are not included among prognostic factors in international breast cancer guidelines. However, some researchers suggest that the total size of all tumor foci may be a more accurate predictor of tumor behavior than the size of the largest focus. Additionally, the presence of multiple foci may indicate distinct tumor biology and has been associated with higher rates of metastatic axillary lymph node [mALN] involvement [38,39].

A recent study comparing the histological tumor type and grade of invasive tumor foci in multifocal/multicentric breast carcinoma with corresponding mALN foci revealed that the morphological features of mALN correspond to the histological tumor type with the highest grade and the most unfavorable prognosis. The tumor type associated with mALN does not necessarily correspond to the largest tumor focus [40]. Furthermore, another study reported that multifocal/multicentric breast cancers tend to exhibit more aggressive behavior, with lower survival rates, higher recurrence rates, and increased lymph node metastasis compared to unifocal breast cancers [41]. Given these findings, preoperative MRI evaluation is crucial for breast cancer patients, as it provides valuable information beyond the primary tumor’s structure. MRI can assess tumor morphology, number, and distribution, enabling clinicians to make more informed and personalized treatment decisions.

### Limitations

There are some limitations in our study. This is a single-center, retrospective study with arelatively low number of cases. MRI images were reviewed by a single radiologist experienced in breast imaging. Observer variability was not assessed, and the study’s single-center design, evaluation using a single device, workstation, and also single-reader bias may affect the generalizability of the findings. Morphological characteristics, kinetic contrast parameters, and ADC values in diffusion imaging were not assessed. Additionally, the presence of multiple lesions and tumor size were evaluated based on MRI findings without comparison to pathological data, because some patients received neoadjuvant chemotherapy, making this comparison impossible. MRI images were assessed without knowledge of clinical, other radiological, and pathological findings. However, some cases had highly suspicious lymph nodes on MRI, which could have affected blind assessment.

## 5. Conclusions

Our findings suggest that the presence of edema and multiple tumor foci in preoperative MRI could be independent prognostic factors for mALN in breast cancer and could be used as prognostic imaging biomarkers. Further research with larger studies is needed to support our findings. Machine learning would be important also in this aspect, with its ability to objectively evaluate many features, some of which are not detectable by the human eye.

## Figures and Tables

**Table 1:** Comparison of clinical characteristics, pathologic findings and MRI findings of patients according to the presence or absence of metastatic ALN.

Variables	Metastatic ALN (−) *n* = 35	Metastatic ALN(+) *n* = 88	*p* Value
**Age (IQR)**	48 (41–63)	45 (39–54)	0.042 *
**Distance from skin (IQR) (mm)**	14 (10–20)	12 (5–18)	0.36
**Lesion Side (right vs. left) *n* (%)**	13 (37.1)	51 (58)	0.037 *
**MRI**			
**Number of lesions *n* (%)**			0.001 *
**Single**	26 (74.3)	37 (42.0)	
**Multifocal**	6 (17.1)	22 (25.0)	
**Multicentric**	2 (5.7)	21 (23.9)	
**Diffuse**	1 (2.9)	8 (9.1)	
**Tumor size in MRI (IQR) (mm)**	20 (17–30)	32 (21–43)	<0.001 *
**Necrosis** ***n* (%)**	5 (14.3)	28 (31.8)	0.05 *
**Diffusion restriction *n* (%)**	26 (74.3)	75 (84.2)	0.13
**Presence of edema *n* (%)**			<0.001 *
**None**	17 (48.6)	14 (15.9)	
**Peritumoral edema**	14 (40.0)	43 (48.9)	
**Diffuse edema**	4 (11.4)	31 (35.2)	
**Background enhancement *n* (%)**			0.30
**Minimal**	4 (11.4)	4 (4.5)	
**Mild**	6 (17.1)	24 (27.3)	
**Moderate**	20 (57.1)	35 (39.8)	
**Marked**	5 (14.3)	25 (28.4)	
**Fibroglandular tissue** ***n*** **(%)**			0.36
**Type A**	2 (5.7)	1 (1.1)	
**Type B**	11 (31.4)	27 (30.7)	
**Type C**	11 (31.4)	27 (30.7)	
**Type** **D**	11 (31.4)	33 (37.5)	
**Ki *n* (%)**	15 (10–25)	30 (15–43)	0.002 *
**Molecular subtype *n* (%)**			0.016 *
**Luminal A**	14 (40.0)	17 (19.3)	
**Luminal B**	17 (48.6)	46 (52.3)	
**Her-2 enriched**	3 (8.6)	13 (14.8)	
**Triple negative**	1 (2.9)	12 (13.6)	

IQR, interquartile range; MRI, magnetic resonance imaging, * Criteria with statistical significance.

**Table 2:** Distribution of anatomical localization of mass lesions in both breasts.

	Right Breast *n* = 64	Left Breast *n* = 59	*p* Value
**Quadrant 1 *n* (%)**			0.58
**Inner**	11 (17.2)	15 (25.4)	
**Outer**	38 (59.4)	31 (52.5)	
**Central**	15 (23.4)	13 (22.0)	
**Quadtrant 2 *n* (%)**			0.44
**Upper**	34 (53.1)	24 (40.7)	
**Lower**	9 (14.1)	14 (23.7)	
**Central**	21 (32.8)	21 (35.6)	
**Depth *n* (%)**			0.35
**Anterior**	12 (18.8)	12 (20.3)	
**Central**	22 (34.4)	18 (30.5)	
**Posterior**	18 (28.1)	20 (33.9)	
**Extensive**	12 (18.8)	9 (15.3)	

**Table 3:** Univariate and multivariate logistic regression analyses to identify variables associated with the presence of metastatic ALN.

Variables	Univariate			Multivariate		
	Unadjusted OR	%95 CI	*p* Value	Adjusted OR	%95 CI	*p* Value
**Age**	0.96	0.93–0.99	0.023	0.95	0.92–1.01	0.051
**Presence of edema (none vs. perilesional-diffuse)**	3.19	1.71–5.95	<0.001	2.46	1.11–5.48	0.027
**Number of lesions (single vs. multicentric-multifocal-diffuse)**	2.31	1.34–3.98	0.003	1.86	1.01–3.43	0.046
**Molecular subtype**	2.05	1.19–3.51	0.009	1.97	0.89–2.48	0.12
**Tumor size (MRI, mm)**	1.05	1.01–1.09	0.004	1.03	0.97–1.06	0.23
**Kİ-67**	1.02	1.01–1.04	0.022	1.02	1.00–1.04	0.62
**Side (right vs. left)**	0.42	0.19–0.96	0.039	0.54	0.26–1.18	0.42

CI, confidence interval; MRI, magnetic resonance imaging; OR, odds ratio.

## Data Availability

The data presented in this study are available on request from the corresponding author. The data are not publicly available due to local policies.
